# Cdk5-dependent rapid formation and stabilization of dendritic spines by corticotropin-releasing factor

**DOI:** 10.1038/s41398-024-02749-7

**Published:** 2024-01-17

**Authors:** Dorien Vandael, Katlijn Vints, Pieter Baatsen, Małgorzata A. Śliwińska, Sergio Gabarre, Lies De Groef, Lieve Moons, Vasily Rybakin, Natalia V. Gounko

**Affiliations:** 1 VIB-KU Leuven Center for Brain & Disease Research, Electron Microscopy Platform & VIB-Bioimaging Core, O&N5 Herestraat 49 box 602, 3000 Leuven, Belgium; 2 KU Leuven Department of Neurosciences, Leuven Brain Institute, O&N5 Herestraat 49 box 602, 3000 Leuven, Belgium; 3 KU Leuven, Leuven Brain Institute, Department of Biology, Animal Physiology and Neurobiology Division, Naamsestraat 61 box 2464, 3000 Leuven, Belgium; 4 National University of Singapore, Department of Microbiology and Immunology, Yng Loo Lin School of Medicine, and Immunology Program, 5 Science Drive 2, Blk MD4, 117545 Singapore, Singapore

**Keywords:** Molecular neuroscience, Psychiatric disorders

## Abstract

The neuropeptide corticotropin-releasing factor (CRF) exerts a pivotal role in modulating neuronal activity in the mammalian brain. The effects of CRF exhibit notable variations, depending on factors such as duration of exposure, concentration, and anatomical location. In the CA1 region of the hippocampus, the impact of CRF is dichotomous: chronic exposure to CRF impairs synapse formation and dendritic integrity, whereas brief exposure enhances synapse formation and plasticity. In the current study, we demonstrate long-term effects of acute CRF on the density and stability of mature mushroom spines ex vivo. We establish that both CRF receptors are present in this hippocampal region, and we pinpoint their precise subcellular localization within synapses by electron microscopy. Furthermore, both in vivo and ex vivo data collectively demonstrate that a transient surge of CRF in the CA1 activates the cyclin-dependent kinase 5 (Cdk5)-pathway. This activation leads to a notable augmentation in CRF-dependent spine formation. Overall, these data suggest that upon acute release of CRF in the CA1-SR synapse, both CRF-Rs can be activated and promote synaptic plasticity via activating different downstream signaling pathways, such as the Cdk5-pathway.

## Introduction

Synapse formation and neuronal networks are built and/or strengthened by exogenous factors such as learning and stress [1]. Neuromodulators exert diverse effects on the formation and pruning of synaptic connections [2, 3]. Among these agents, corticotropin-releasing factor (CRF), a neuromodulator intricately linked to synaptic plasticity, plays a central role in the hypothalamic–pituitary–adrenal axis (HPA), which is a stress axis [4, 5]. With the onset of stress, CRF is released in the paraventricular nucleus of the hypothalamus. The HPA axis operates as a feedback-regulating system in which CRF release leads to the synthesis of glucocorticoids [5]. These glucocorticoids suppress the secretion of CRF upon reaching the hypothalamus and higher brain regions [5, 6]. Beyond its role in the HPA axis, CRF is endogenously expressed in discrete regions of the brain, where it regulates dendrite development and maturation, synaptogenesis, and the integration of adult-born neurons into neuronal circuits [7–9]. In chronic stress, characterized by prolonged exposure or elevated CRF concentrations, there is a decline in spine density and dendritic complexity [10]. However, our recent findings also underscored the significance of CRF in acute stress situations [11]. CRF modulates excitatory transmission within specific neurons via two receptors: CRF-R1 and CRF-R2 [12, 13]. CRF-R1 and CRF-R2 are classified as type B G protein-coupled receptors. Each CRF-R originates from discrete genes and exhibits multiple splice variants [14]. CRF-R1 and CRF-R2 are ubiquitously expressed in both central and peripheral tissues with a 70% amino acid sequence similarity in their transmembrane and intracellular domains [15, 16]. CRF-Rs can be activated by various stimuli, including stress, CRF, and CRF-related peptides such as urocortin 1, urocortin 2 (or stresscopin-related peptide), and urocortin 3 (or stresscopin). Binding of CRF-R agonits with the extracellular domains of CRF-R1 and R2 induces structural alterations in these receptors, ultimately activating G-proteins and initiating downstream signaling cascades [17].

The distribution of CRF and CRF-Rs varies between specific areas of the brain. Due to CRF’s neuromodulatory nature it can induce volumetric remote actions, therefore the localization of CRF-Rs can differ from its release sites [18]. While previous studies have reported the presence of CRF in a subpopulation of GABAergic interneurons in the hippocampus and CRF-Rs in excitatory synapses of the pyramidal cells (PCs), precise subcellular localization remains debated. Previous reports documented the presence of CRF-R2 in the hippocampus but in lower amounts than CRF-R1 [18, 19]. Interestingly, acute CRF exposure demonstrates an increase in calcium-dependent vesicular release and an increase in the active zone [11], which are modifications happening in the presynaptic compartment. In addition, both CRF-Rs influence spine type, and its activation enhances long-term plasticity, as seen in long-term potentiation (LTP) measurements documented in previous observations [11]. However, CRF-R2 expression and its role in the hippocampus remain unclear. Using different labeling techniques and electron microscopy, we demonstrate the exact localization of CRF, CRF-R1, and CRF-R2 in the CA1-SR and identify long-term effects of acute CRF exposure, such as an increase in mature spines with enduring stability over hours. Notably, both CRF-Rs are present in the hippocampus, regulating CRF actions in synapse morphology and function. The molecular mechanisms behind CRF peptide’s regulation of spine density, although not fully elucidated, likely involve changes in the actin cytoskeleton. One of the promising candidates in the CRF-dependent regulation of spine formation is Cdk5, a serine/threonine kinase vital for neuronal development, spine formation, learning, and memory [20, 21, 22].

This study utilizes two-photon ex vivo dendritic spine imaging and in vivo stereotactic injections to reveal the transient and stabilizing effects of acute CRF in the CA1-SR synapses. It further showcases the presence of CRF-R1 and CRF-R2 and localization in CA1 synapses, in driving CRF-induced spine increase. Altogether, this work indicates CRF’s role in spine formation, stabilization, and cytoskeletal rearrangements through the activation of the Cdk5 pathway.

## Materials and methods

### Animal experiments

All animal experiments were approved by the KU Leuven Ethical Animal Welfare Committee (protocol P019/2017 and P070/2022). Mice were housed in a pathogen-free facility under standard housing conditions. All experiments were performed according to the Animal Welfare Committee guidelines of the KU Leuven, Belgium.

### Mouse lines

Acute cell fillings, electrophysiology, biotin, and immuno-labeling were performed in P18-P20 old male C57BL/6 mice (Jackson Laboratory). Spine visualization was performed in P18-P20 old male mice of the Thy1-YFP-H line (B6.Cg-Tg(Thy1-YFP)HJrs/J - JAX 003782, Jackson Laboratory).

### Acute slice preparation

Acute slices of P18-P20 Thy1-YFP-H or C57BL/6 mice were prepared. Briefly, mice were anesthetized with isoflurane and rapidly decapitated. After decapitation, the brain was quickly removed and placed into an ice-cold sucrose-based cutting solution consisting of: 83 mM NaCl, 22 mM glucose, 72 mM sucrose, 2.5 mM KCl, 1 mM NaH_2_PO_4_, 0.5 mM CaCl_2_, 3.3 mM MgSO_4_, 26.2 mM NaHCO_3_ (Sigma-Aldrich). Coronal slices (300 µm) were cut using a vibratome (VT1200, Leica Microsystems) in a cutting solution (see above). These slices were allowed to recover for 35 min at 34 °C and were maintained at room temperature (RT) within the same solution for at least 30 min prior to use.

### Imaging, visualization, and analysis of spine types

Acute slices of Thy1-YFP-H were used to see morphological effects of CRF. Dendritic segments of CA1 PCs were imaged with a two-photon system (VIVO 2-Photon platform, Intelligent Imaging Innovations GmbH) using a 20X objective. Imaging in artificial cerebrospinal fluid (aCSF) was used as a control condition, and a z-stack was defined with a step size of 0.01 µm with 4 frames per plane. The maximum input power was set between 17% and 25%, depending on the expression level and the signal background in the brain section. After capturing the control condition, the imaging solution was changed to aCSF with 100 nM CRF. For each animal, sections for each different time point were gathered. After 20 min, a new z-stack was obtained with the same parameters, and then the medium was changed back into the control aCSF solution. Wash out time frames were obtained 30 min after CRF incubation to see changes in spine dynamics. In parallel, for spine type quantification: control (CTRL) and CRF and different wash out times of 30, 60, and 120 min treated slices were fixed in 4% paraformaldehyde (PFA, EMS) PFA and 2% sucrose in 0.1 M phosphate buffer (PB, EMS) (pH 7.4) at 4 °C overnight. Slices were then washed three times with 0.1 M PB solution, and coverslips were mounted using a mounting medium with 4′,6-diamidino-2-phenylindole (DAPI, Vectashield). Secondary and tertiary CA1 proximal dendrites were imaged on a Zeiss ELYRA S.1 structured illumination microscope. Z-stack images of dendritic structures were captured using a 63X oil objective with a step size of 0.025 µm. Subsequent quantification was performed using ImageJ (NIH). We categorized five distinct spine types. Mushroom spines: the spines possess a spine head exceeding 0.5 µm. Stubby spines: characterized by a length shorter than 1.0 µm and a spine head diameter larger than the spine length. Thin spines: spines with a length shorter than 1.0 µm and a spine head diameter shorter than the spine length. Long thin spines: length between 1.0 and 1.5 µm. Filopodia: spines with a spine length exceeding 1.5 µm. By implementation of this classification, we gained insights into the morphology of the dendritic spines in the CA1.

### Imaging Cdk5-dependent spine formation, ex vivo and in vivo

For ex vivo spine analysis, C57BL/6 mice were used as previously described [13]. Briefly, acute slices were prepared as described above. After the last step of recovery at RT for 30 min, slices were transferred into aCSF. Glass borosilicate recording pipettes (resistance 3.5–5.5 MΩ) were filled with 10 mM Alexa 568 (Life Technologies). Whole-cell configuration was used to fill CA1 PCs for 10–15 min in CTRL slices. Slices were incubated with 100 nM CRF added to the aCSF for 20 min. Hence, slices were incubated 10 min prior to filling with Alexa 568. Treatment with roscovitine (ROS, Tocris) was carried out by directly adding the compound to the aCSF at least 20 min before reaching whole cell mode. For the condition with ROS combined with CRF, CRF was added 10 min after slices were exposed to ROS. Sections were fixed with 4% PFA and 2% sucrose (EMS) in 0.1 MPB (pH 7.4) at 4 °C overnight. For in vivo spine analysis, Thy1-YFP-H mice were injected intraperitoneally (IP) with 25 mg/kg ROS 30 min before stereotactic injections of 100 nM CRF in CA1 PCs. Mice were anesthetized with isoflurane and placed in a stereotactic frame with sustained anesthesia during the injection. Aliquots (300 nL) of 100 nM CRF were unilaterally injected at a rate of 10 nL/sec with a Nanoject II Auto-Nanoliter Injector (Drummond) using stereotactic coordinates: AP-2 mm, ML-1.8 mm, D-1.5 mm. The non-injected hemisphere was used as a control. Animals were perfused with 4% PFA in 0.1 M PB, 20 min after the initial injection. Slices were generated, imaged, and quantified as described above.

### Sample preparation for CRF and CRF-Rs labeling

*For pre-embedding CRF-R2 labeling*, 250 µm ex vivo acute slices were used, prepared as described above. Control slices without any treatment and slices treated for 30 min with 150 nM CRF-R2 blocker coupled with biotin (GenScript). After treatment, brain sections were fixed overnight at 4 °C using a solution of 4% PFA and 0.01% glutaraldehyde (GA, EMS) in 0.1 M PB (pH 7.4). Subsequently, coronal sections were washed by 0.1 M PB, followed by blocking in 0.1% sodium borohydride (Sigma-Aldrich) in 0.1 M PB for 30 min on ice, washed again with 0.1 M PB, followed by a second blocking step in 0.01% glycine (Sigma-Aldrich), 0.01% lysine (Sigma-Aldrich), 0.1% cold water fish gelatin (CWFG, EMS), 20% BSA (Sigma-Aldrich) and 0.01% triton (EMS) in 0.1 M PB for 2 h. Afterwards, some sections were incubated with Alexa 555 streptavidin (1:1000, Nanoprobes) overnight at 4 °C and others were taken for DAB (diaminobenzidine)/GSSP (gold-substituted silver peroxidase) immunolabeling for CRF-R2. The next day, sections were washed with 0.1 M PB and were mounted on microscopic slides using 0.1 M PB containing DAPI (1:2000). Sections were imaged with a confocal Nikon C2 with a 20X objective to verify the labeling before proceeding with the embedding. For antibody specificity, some section we treated same way as described before but without using primary antibodies, and some with different secondary antibodies.

To visualize DAB/GSSP immunoreactivity, we used the avidin–biotin complex (ABC) method. The ABC complex (Vector Laboratories) is prepared strictly in accordance to the manufacturer’s instructions. The initial steps of the protocol involve series of washing steps: two cycles of immersion in 0.1 M PB, followed by three immersions in PB containing 0.01% Triton X-100 for 10 min each on ice. Afterwards, Slices were incubated with the ABC complex over the course of 1 h at RT. To visualize the immunoreactivity, we incubated the sections with a solution containing 5 mg of 3.3′-DAB (EMS) and 0.03% hydrogen peroxide dissolved in 10 ml of 0.1 M PB solution for 10 min at RT. The termination of the reaction was induced by immersing the section in cold 0.1 M PB, whereafter the slices were fixed with 2% GA in 0.1 M PB for 1 h, on ice. Following this, slices were subsequently washed four cycles in 0.1 M PB followed by three cycles in 2% sodium acetate (EMS) diluted in distilled water for 10 min each on ice and stored overnight in 10% sodium thioglycolate in distilled water (Sigma-Aldrich) maintained at a temperature of 4 °C. The labeling was enhanced by the implementation of the GSSP method [22]. Post GSSP reaction, the sections were washed in 0.1 M sodium cacodylate (EMS) buffer involving four cycles of 10 min, on ice. The slices were subjected to staining with 1% osmium tetroxide (EMS) and 1.5% potassium ferrocyanide (EMS) in 0.1 M sodium cacodylate trihydrate (EMS) buffer for 15 min. This was followed by five sequential washing steps in distilled water, each for 7 min. Subsequently to the washes, sections underwent a dehydration process involving ascending ethanol solution steps (30%, 50%, 70%, 80%, 95%), 10 min per step, at 4 °C. Additionally, the samples were subjected for two 15 min treatments each with absolute ethanol at RT and infiltrated with medium Epon 812/ethanol mixtures overnight. The following day, sections were flat-embedded using a medium composition of Epon 812 (EMS) between two microscopic slides and ACLAR film (EMS) and subsequently polymerized for 2 days at 60 °C.

*For CRF and CRF-R1 post-embedding immunogold labeling*, C57BL/6Jax mice were perfused with 4% PFA, 0.5% GA in 0.1 M PB (pH 7.4) and stored in the same fixative overnight. The next day, after three washes with 0.1 M PB for 10 min, vibratome sections of 80 µm were cut.

For CLEM-imaging, hippocampal sections were placed in a solution of 20% BSA diluted in 0,1 M PB. Regions of interest in the CA1-stratum radiatum (SR) were punched out from these sections and frozen in 3 mm carriers with a high-pressure freezer (HPF ICE, Leica Microsystems). Frozen samples were quick freeze-substituted and embedded according to our protocol [23] using EM AFS2 apparatus (Leica Microsystems). Subsequently, 70 nm sections were cut with an ultramicrotome (Ultracut S, Leica Microsystems). For CRF and CRF-R1 post-embedding labeling, the sections were collected onto a formvar film-coated 100-mesh nickel grid (EMS). For CRF-R2 imaging, the sections were collected on formvar film-coated 75 mesh copper grids (EMS). For CRF-R2 imaging, the sections were collected on formvar film-coated 75 mesh copper grids (EMS). For DAB/GSSP CRF-R2 labeling, the 70 nm sections were collected onto a formvar film-coated one slot grids (EMS).

For labeling of CRF and CRF-R1, we used the following antibodies and dilution ratios in our studies: primary 1:1000 rabbit anti-CRF (Salk), 1:200 rabbit anti-CRF-R1 (ACR-050, Alomone labs), and secondary 1:30 donkey anti-rabbit 6 nm gold (Aurion). The grids were etched for 2 s on 15% sodium hydroxide (EMS) droplets. After washing with distilled water, they were incubated on droplets of tris buffer (EMS) with 0.01% triton, 0.2% sodium borohydride and 0.05 M glycine for 15 min. Next, grids were washed with tris buffer containing 0.01% triton followed by incubation on droplets of tris buffer with 0.05 M glycine, 0.1% CWFG, 20% BSA, 10% goat serum (Aurion) and 0.01% triton for 1 h. Afterwards, grids were placed on droplets containing the primary antibodies in the same incubation buffer overnight at 4 °C in a humidity chamber. The next day, washes with tris buffer containing 0.01% triton were performed before incubation with secondary antibody in same incubation buffer for 2 h at RT. After the final washing with tris buffer and distilled water, the grids were fixed with 1% GA in distilled water for 5 min and contrasted with 2% uranyl acetate (EMS) in distilled water for 8 min. EM observation was done with a transmission EM (TEM, JEM1400 Jeol) equipped with an SIS Quemesa camera (Olympus) operating at 80 kV.

For CRF-R2 imaging, after dipping copper grids with sections in 20% BSA in distilled water, grids were washed with distilled water two times. Followed by placing them in a droplet of distilled water with DAPI (1:2000) on a coverslip (18 mm ϕ) for imaging in a metal holder with a confocal Nikon C2. The region of interest was first imaged with a 20X objective to make an overview and with a 60X objective for higher resolution. After confocal imaging, grids were allowed to dry and studied by TEM. After detecting the same nuclei, a correlating overview image was taken at 1.5 k magnification together with a high-resolution image at 15 k magnification (pixel size 0.95 nm).

### Long-term ex vivo potentiation recordings with multi electrode array (MEA)

Parasagittal acute slices (300 μm) were prepared from C57BL/6Jax mice and used for recording field excitatory postsynaptic potentials (fEPSPs) using commercially available multi-electrode arrays (MEAs) in an 8 × 8 layout (MEA2100, Multi-Channel Systems). To maintain a conductive environment, the recording chamber was perfused with aCSF and maintained at a constant temperature of 32 °C. A slice grid was put on the surface of the slices to assure immobilization and optimal electrode contact. Data streams were sampled at a rate of 10 kHz. For each slice, a single electrode located underneath the Schaffer collateral (SC) pathway was visually selected for stimulation. Biphasic, constant voltage pulses (100 µs pulse width) were applied to evoke fEPSPs from the SCs in the CA1. Afterward establishing stable fEPSP signals (after approximately 30 min), an input/output curve was generated using stimulation intensities from 0.5 to 2.750 V (step size of 0.25 V), each applied twice and with an interval of 30–120 s. The stimulus intensity eliciting 35% of the maximal fEPSP amplitude was used for further stimulation.

Next, we recorded baseline fEPSPs for approximately 25 min (3 stimulations, 15 s apart, and every 3 min). For CRF conditions, after 5 min of baseline, we switched to aCSF with 100 nM CRF, recorded for 15 min, and switched back to pure aCSF, which normalized a stable baseline comparable to that seen before CRF application. After reestablishing a stable baseline, we applied three trains of high-frequency stimulation at 100 Hz (100 stimuli at 100 Hz) with 5-min intervals.

### Chemicals and treatments

Antisauvagine-30 (CRF-R2 blocker, GenScript) coupled with biotin—150 nM, CRF—100 nM (Bachem), NBI 27914 (NBI, CRF-R1 blocker)—1.2 µM (Tocris), Roscovitin—25 µM ex vivo or 25 mg/kg for system injections (Tocris). All compounds were dissolved in DMSO prior to dilution into appropriate aqueous buffers/solutions. In case of CRF exposure, 100 nM of CRF was added for 20 min.

### Quantification and statistical analysis

Data analysis was carried out in ImageJ (NIH), Mini Analysis (Synaptosoft), Multi Channel analyzer software (Multi Channel Systems), and Excel (Microsoft). Data statistics were calculated in GraphPad Prism 8 (GraphPad software). Spine analyses were performed blindly to ensure unbiased assessments. We did not calculate sample size for ensuring adequate power or randomization of the samples. Animals and brain sections with a deteriorated general health were systematically excluded from the study to maintain data integrity.

**Fig. 1:**
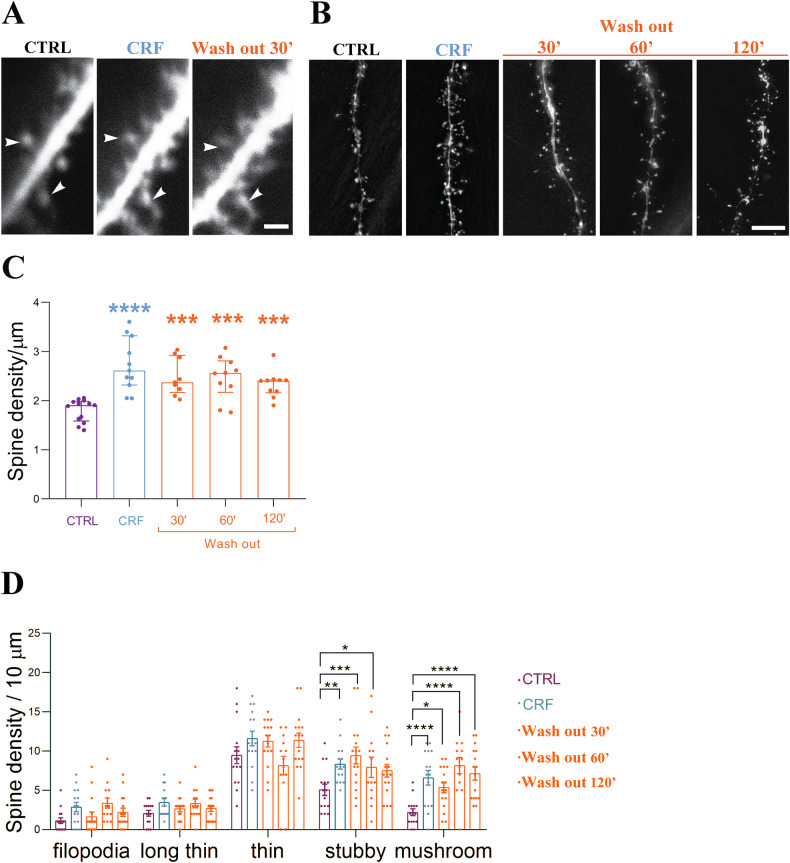
Acute CRF leads to hours-long spine growth ex vivo. **A** Representative 2 photon images from the same CA1 proximal dendrite in control (CTRL) condition, after incubation in 100 nM CRF for 30 min, and after washing out for 30 min. **B** Representative images of dendritic segments of CTRL, CRF incubation (100 nM for 20 min), and 30, 60, and 120 min of wash out. **C** Spine density across the different experimental groups. **D** spine type distribution across the different experimental groups. The data are represented as the mean ± SEM. CTRL (*N* = 4 animals, *n* = 13 cells, *d* = 21 dendrites), CRF (*N* = 4, *n* = 11, *d* = 21), 30 min wash out (*N* = 4, *n* = 10, *d* = 17), 60 min wash out (*N* = 4, *n* = 11, *d* = 21), and 120 min wash out (*N* = 4, *n* = 12, *d* = 27), Statistical analysis conducted using a one-way ANOVA with Dunnett’s Multiple Comparison test. Significance levels are indicated by asterisks: ***p < 0.0005, ****p < 0.0001. **D** Spine type distribution across the different experimental groups. The data are represented as the mean ± SEM. CTRL (*N* = 4 animals, *n* = 13 cells, *d* = 21 dendrites), CRF (*N* = 4, *n* = 11, *d* = 21), 30 min wash out (*N* = 4, *n* = 10, *d* = 17), 60 min wash out (*N* = 4, *n* = 11, *d* = 21), and 120 min wash out (*N* = 4, *n* = 12, *d* = 27), Statistical analysis conducted using a two way ANOVA with Dunnett’s Multiple Comparison test. Significance levels are indicated by asterisks: *p < 0.05, **p < 0.001, ***p < 0.0005, ****p < 0.0001. Scale bar = 2 µm (**A**), 5 µm (**B**).

For statistical data analysis, initially, we evaluated the adjustment of quantitative sample distributions to a theoretical normal distribution using the D’Agostino-Pearson test. For multiple group comparisons with a non-normal distribution: Kruskal-Wallis analysis of variance (ANOVA) was conducted, followed by Dunn’s multiple comparison test for post hoc analysis. For multiple-group comparisons with a normal distribution, One-way ANOVA was conducted, followed by Dunnett’s Multiple Comparison. In cases of two factors such as quantification of different spine type over time into the different experimental groups (Fig. 1D), two-way ANOVA followed by Dunnett’s multiple comparisons was conducted. Normal distributions are represented as the mean with the standard error of the mean (±SEM) while non-normal distributions are represented as the median with the interquartile range. All results were evaluated at a 5% significance level. Comprehensive details regarding sample size and statistical tests (including p-values) used for each comparison are in detail described in the corresponding figure legends.

## Results

### CRF transiently and robustly increases spine density

To observe spine formation and turnover, we performed two-photon imaging of ex-vivo acute slices prepared from Thy1-YFP mice. By visualizing the same dendritic segment, we found that there is an increase in spine density after treatment with 100 nM CRF for 20 min (Fig. 1A). We could determine that there is an increase in newly formed spines, and not an increase in spine turnover, because of the stable representation of the existing spines (Fig. 1A, white arrows) with no visible spine retraction.

To answer the question of the long-lasting effects of CRF, we observed spine density dynamics over longer periods and quantified spine densities at different time points (30 min to 2 h) after CRF exposure. We observed an increase in total spine density after CRF incubation (Fig. 1C) and more specifically an increase in the more stable and mature spine types as mushroom and stubby spines (Fig. 1D), as previously described [11]. Spine density increased and stabilized after 30 min of washing out and remained constant over the period of 2 h (Fig. 1C). Notably, the mature spine type numbers maintained significantly increased (Fig. 1D) [11] suggesting that CRF evokes a spine increase and formation of more stable spine types, lasting for a relatively long period of time.

### Localization of CRF and CRF-Rs in the synapse

**Fig. 2:**
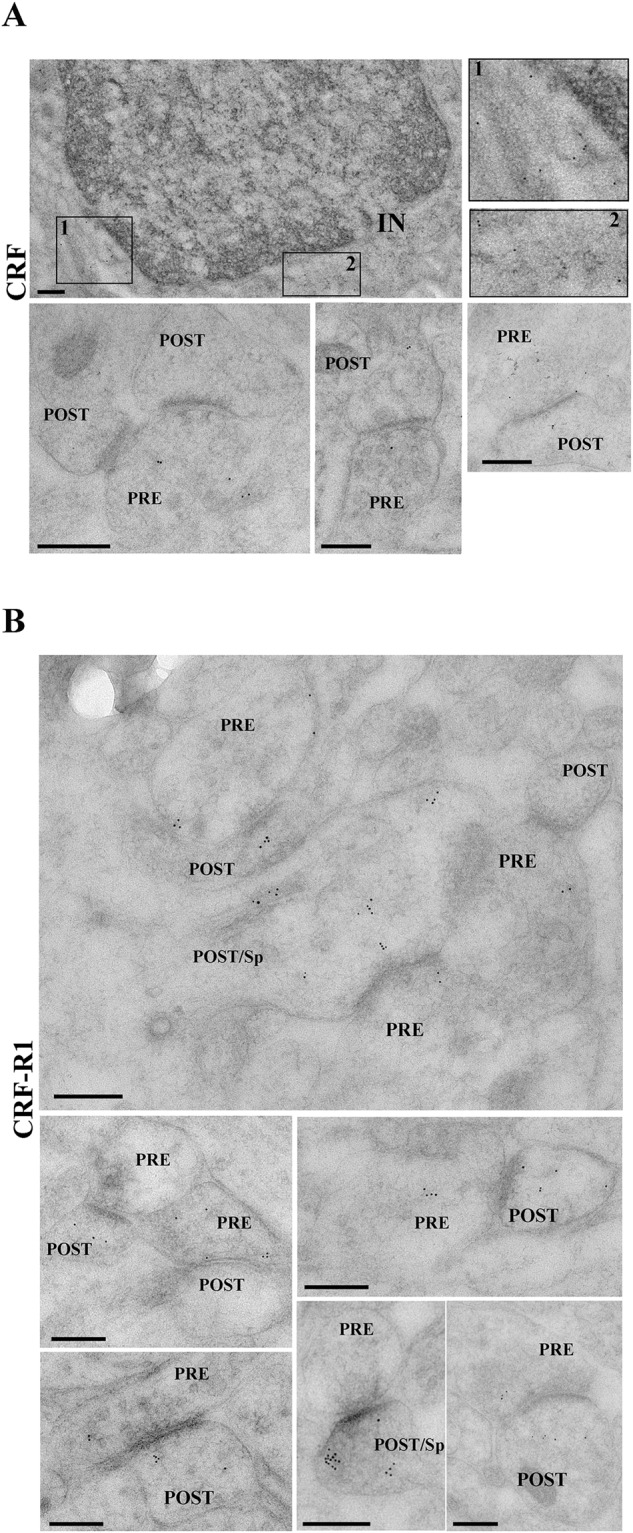
Presence CRF and CRF-R1 in the SC-CA1 hippocampal synapse. **A** TEM images of CA1-SR area. Immunogold labeling for CRF is present in the interneuronal (IN, top) and excitatory (bottom) synapses at both parts of the synaptic boutons (PRE: presynaptic part of the synapse, POST: postsynaptic part of synapse, POST/Sp: spines - postsynaptic part of synapse), but labeling is predominantly located in the presynaptic compartment. **B** TEM images of CRF-R1 immunogold labeling. CRF-R1 appears at both synaptic sites in excitatory synapses. Scale bar = 200 nm.

First, we looked at the distribution of CRF and CRF-R1 at the subcellular level. We found that CRF immunoreactivity was widely present throughout the CA1-SR region. As positive control, we validated CRF-R1 presence in the cerebellum (data not shown), where it can be observed at the Golgi apparatus of the Purkinje cells, climbing fibers synapses, and soma of interneurons. CRF labeling appears to be in the hippocampal interneurons (Fig. 2A, top) and excitatory synapses in this area in both the pre- and postsynaptic compartments (Fig. 2A, bottom). However, CRF labeling appears to be more intense in the presynaptic parts of the synapses (Fig. 2A, bottom). Next, we looked at CRF-R1 labeling. EM gold labeling showed that positive CRF-R1 profiles are in the excitatory synapses in CA1-SR (Fig. 2B). CRF-R1 is present in both parts of the synaptic compartments and not only in the postsynaptic compartment on the spine head. We have used double labeling of either CRF or CRF-R1 with the presynaptic marker VGLUT1 (Supplementary Fig. 1), to provide complementary information on their ultrastructural localization in synapses. Similarly, to findings shown in Fig. 2B, we observed the majority of CRF and CRF-R1 staining in presynaptic structures. In the presynapse, CRF-R1 can potentially induce presynaptic calcium-dependent effects, which is confirmed by the observations of an increased frequency of spontaneous activity and a rise in paired pulse facilitation in the presence of CRF [11].

**Fig. 3:**
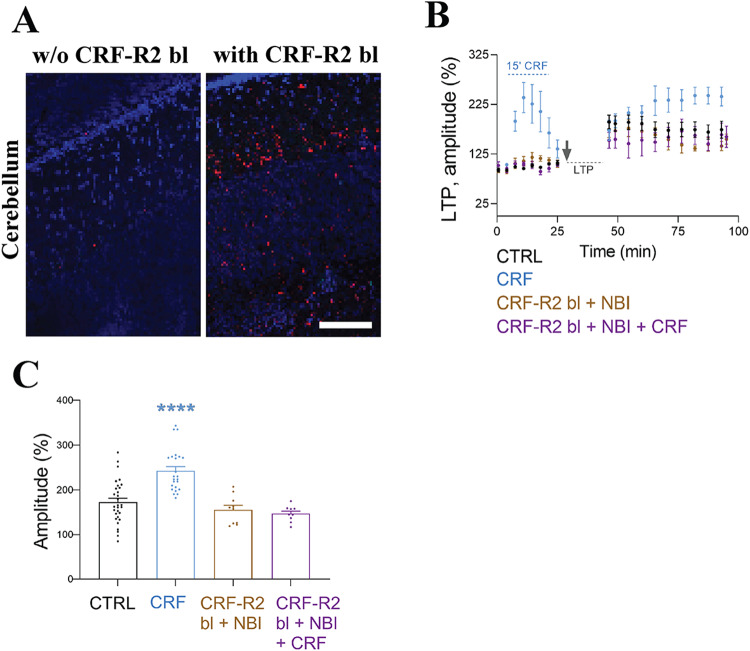
Biotinylated antisauvagnine-30 is a highly specific tool for CRF-R2 detection in situ. **A** Light microscopy staining for biotin-streptavidin, left the ex vivo slices without CRF-R2 blocker vs right with CRF-R2 blocker (150 nM) for 30 min. In red CRF-R2 positive cells in cerebellum. Conformation functional binding of CRF-R2 blocker coupled with biotin (**B**) LTP induction in combination with CRF-R1 blocker (NBI, 1.2 μM) with CRF-R2 blocker (CRF-R2 bl) Antisauvagine-30 coupled with biotin (150 nM) in the presence and absence of CRF (100 nM, 15 min). **C** The use of both CRF-Rs blockers perturbs CRF-dependent increase in LTP as shown before [15], confirming that CRF-R2 blocker coupling with biotin is functional and binds to CRF-R2, preventing enhancement of LTP. Data are represented as the mean ± SEM. Control (CTRL) (*N* = 10 animals), CRF (*N* = 8), CRF-R2 bl + NBI (*N* = 4), and CRF-R2 bl + NBI (*N* = 4), compared b**y** one-way ANOVA with Dunnett’s Multiple Comparison (*F* = 18.19). Significance levels are indicated by asterisks: ****p < 0.0001. Scale bar = 100 µm.

**Fig. 4:**
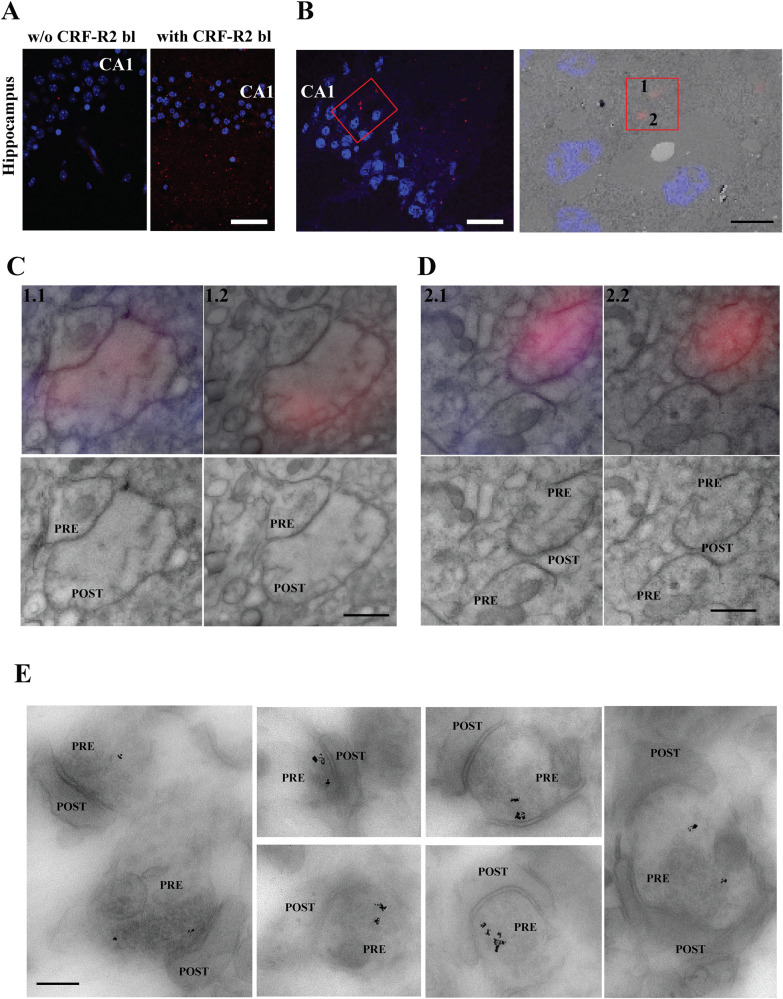
Presence CRF-R2 in the SC-CA1 hippocampal synapse. **A** Light microscopy staining for biotin-streptavidin, left the ex vivo slices without CRF-R2 blocker (CRF-R2 bl) vs right with CRF-R2 blocker (150 nM) for 30 min. In red, CRF-R2 positive cells in CA1-SR region of hippocampus. **B** Two red dots in red box, biotin-streptavidin labeling for CRF-R2 blocker at LM are found at EM level - same dots indicated by numbers 1, and 2 at right image. **C** More detailed observation at high magnification on the same two dotes as displayed in Fig. 5B. Images of two immediately adjacent consecutive 150 nm sections without any additional post-staining, imaged at higher magnifications within FM mode with Plan Apo VC 100x lens and with TEM. CRF-R2 appears at presynaptic sites in excitatory synapse boutons (PRE: presynaptic part of the synapse, POST: postsynaptic part of synapse). **E** Scale bar = 40 µm (**A**), 19 µm (**B** left) and 5 µm (B right), 500 nm (**C**, **D**), and 250 nm (**E**).

Due to the absence of validated CRF-R2 antibodies suitable for light (LM) or EM, we made use of the ability of the biotinylated CRF-R2 blocker Antisauvagine-30 (Ki = 1.4 nM; CRF-R2 bl), coupled with a biotin to the N-terminal and the C-terminal amidation of the Antisauvagine-30 peptide sequence (GenScript). To validate its efficacy, we subjected the modified CRF-R2 blocker to acute cerebellar (Fig. 3A) and hippocampal (Fig. 4A) slices. Acute cerebellar slices were utilized as a positive benchmark, given the well documented presence of CRF-R2 in the cerebellum [24–26]. Subsequently, we performed a biotin-streptavidin staining for LM (Figs. 3A, 4A), which clearly showed the presence of CRF-R2 in CA1.

In parallel, we performed a LTP-protocol of the SC-CA1 pathway, using MEA extracellular field potential recordings (field excitatory postsynaptic potentials, fEPSPs), following our established procedure [11]. By incorporating our newly modified CRF-R2 blocker coupled with biotin, we managed to successfully reproduce our previous LTP results [11], thus validating its effectiveness (Fig. 3B, C). We further conducted experiments with a combination of both CRF-R1 and CRF-R2 blockers, which completely abolished the acute CRF-dependent LTP enhancement. Therefore, activation of either CRF-R1 or CRF-R2 suffices to induce CRF-dependent enhancement of LTP .

Subsequently, we were able to map the precise localization of CRF-R2 in the CA1-SR (Fig. 4B–E) by using correlative light electron microscopy (CLEM). Firstly, the ex vivo hippocampal slices, treated with modified CRF-R2 blocker coupled with biotin (150 nM) for 30 min were labeled with streptavidin for LM and then EM to localize the CRF-R2 in the CA1-SR. Our investigation definitely confirmed the existence of CRF-R2 in the hippocampus. Furthermore, the use of EM imaging confirmed its localization at the synaptic compartments (Fig. 4B). Since the correlation accuracy can be a complex aspect, we applied the biotinylated CRF-R2 blocker followed by a highly sensitive DAB/GSSP detection protocol [22] to decipher the precise synaptic localization of CRF-R2. As a result, numerous immunopositive synapses were apparent throughout the CA1-SR region, with the reaction products prominently observed in the presynaptic compartment of the excitatory synapses. (Fig. 4E). In summary, our results provide a clear and comprehensive understanding of the precise distribution of CRF-R2 in the CA1-SR region.

### CRF-dependent spine increase requires Cdk5-signaling

**Fig. 5:**
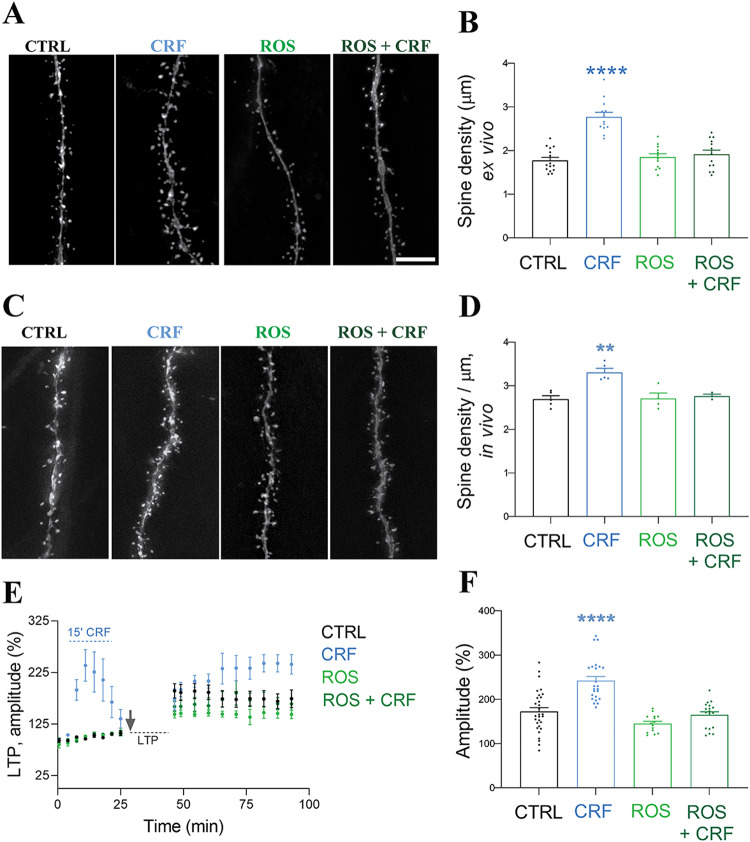
Rapid CRF-dependent changes require Cdk5 activation ex vivo and in vivo. CA1 PCs in ex vivo acute hippocampal slices filled with Alexa 568 dye under control (CTRL) conditions, after 20 min exposure to 100 nM CRF, and with Cdk5 inhibitor ROS (25 µM, 30 min) with or without CRF 100 nM exposure (20 min). Scale bar = 5 µm. **B** Quantification of spine densities as in (**A**). **C** PC1 CA1 dendrites in Thy1-YFP mice with 25 mg/kg ROS with or without stereotactic injection of 100 nM CRF (20 min) into CA1 PCs in vivo. ROS was injected by IP 30 min prior to stereotactic injection of CRF. **D** Quantification of spine densities as in (**C**). **B**: Data are presented as the mean ± SEM. Ex vivo: ROS CTRL (*N* = 3 animals, *n* = 15 cells), CRF (*N* = 3, *n* = 13), ROS (*N* = 3, *n* = 12), and ROS + CRF (*N* = 3, *n* = 13), compared by one-way ANOVA followed by Dunnett’s Multiple Comparison. Significance levels are indicated by asterisks:****p < 0.0001. **D** Data are presented as median with IQR. In vivo: ROS CTRL (*N* = 5 animals), CRF (*N* = 5), ROS (*N* = 4), and ROS + CRF (*N* = 3), compared by Kruskal Wallis test (Kruskal-Wallis’s statistic = 10.22) followed by Dunnett’s Multiple Comparison. Significance levels are indicated by asterisks:**p < 0.005. (E) LTP induction in combination with ROS (25 µM) in the presence and absence of CRF (100 nM, 15 min). **F** ROS completely blocked the CRF-dependent increase in CRF as well as the acute increase in fEPSPs at baseline recordings. Data are represented as the mean ± SEM. CTRL (*N* = 11 animals), CRF (*N* = 8), ROS (*N* = 6), and ROS + CRF (*N* = 6), compared by one-way ANOVA with Dunnett’s Multiple Comparison (*F* = 2.311). Significance levels are indicated by asterisks: ****p < 0.0001. Scale bar = 5 µm.

Next, we asked whether CRF signaling might affect spine formation via activation of the Cdk5 pathway. To explore this, we applied CRF ex vivo (Fig. 5A, B) and in vivo through CRF microinjections (Fig. 5C, D) in combination with the Cdk5 inhibitor roscovitine (ROS), extensively characterized in vivo and in tissue culture [27]. ROS administration at a dosage of 25 mg/kg through intraperitoneal injections (IP) has demonstrated brain-protective effects in vivo [28]. In our experiments, the administration of ROS abolished CRF-induced spine formation in both in vivo and ex vivo. Notably, the application of ROS alone did not affect spine number as compared to control conditions. Electrophysiological recordings of fEPSPs with pre-incubation with CRF and ROS prior to induction of LTP through a high frequency stimulation protocol showed that ROS completely abolished the CRF-dependent increase in amplitude in baseline, acute, and long-term scenarios (Fig. 5E, F). In conclusion, these results strongly indicate the involvement of the Cdk5 pathway in CRF-dependent synaptic alterations.

## Discussion

A complete and thorough investigation is essential to understand individual CRF action in different contexts before addressing its interaction with various neuropeptides and hormones in a time and dose-dependent fashion. Here, we show the effects of CRF in short time windows on the level of PCs in the CA1, resembling the initial stages of the stress response.

CRF actions are mediated by the activation of its two receptors, CRF-R1 and CRF-R2. While prior studies acknowledge the importance of CRF-R1 activation in the hippocampus [18], our research revealed alterations in spine type when CRF-R2 was blocked [11]. Consequently, CRF-dependent LTP enhancement is dependent on both CRF-R1 and CRF-R2 [11]. Nonetheless, additional experiments are imperative to provide deeper insights into the activation of CRF-Rs when there is an acute presence/rise in CRF, along with the presence of other receptor agonists.

Labeling of the CRF-Rs may contribute to knowledge of their presence and function. By immunogold labeling, we visualized the exact localization and precise distribution of CRF and CRF-R1 in the CA1-SR. While CRF displays pre- and postsynaptic effects in the hippocampus [11], CRF-R1 has been exclusively described in the postsynaptic compartment [16, 18, 29]. In contrast, studies in other brain regions have shown the presence of CRF-R1 in both pre- and postsynaptic compartments [30, 31]. The presynaptic localization of CRF-R1 agrees with physiological data observed in other brain regions [32, 33]. Such presynaptic receptors can act as auto receptors [34], which is not an unique phenomenon among neuropeptides.

As for CRF-R2, its presences is closely associated to postsynaptic densities [35]. Although, parallel to our findings, there are reports demonstrating CRF-R2 presence in presynaptic nerve terminals in other brain regions [36, 37]. Here, we show for the first time the presence of CRF-R2 in the hippocampus and its subcellular localization at the synapse within the CA1-SR region.

Depending on which CRF-R is activated by CRF, different molecular signaling pathways can be activated by different G-proteins [38–40].Nevertheless, the molecular mechanisms by which CRF peptides regulate spine density in acute stress and synaptic structural changes in response to the stress are still unclear. We are assuming that the major function of CRF is to regulate the distribution of transmembrane proteins in the postsynaptic membrane, most probably via cytoskeletal changes. For example, previous studies reported that CRF and urocortin (another CRF family peptide) differ in their mechanisms of action on spine density and ultrastructural organization of the pre- and postsynaptic boutons in cerebellar neurons, resulting in functional and morphological phenotypes that are unique to each peptide [4, 41]. In cerebellar slices, extensive redistribution of glutamate receptor delta 2 (GluRδ2) was observed upon application of CRF. The application of urocortin resulted in a significant upregulation of GluRd2 gene expression and protein levels [41]. Hence, it has been hypothesized that the major function of CRF is to regulate the distribution of transmembrane proteins in the postsynaptic membrane, most probably via cytoskeletal changes [42–44].

Our data suggest that CRF-dependent spine changes are Cdk5-dependent (Fig. 5). The active kinase is formed when Cdk5 associates with cofactors p35 or p39 (CDK5R1 and CDK5R2, respectively), and regulates neurotransmitter release by phosphorylating several pre- and postsynaptic proteins [45]. Cdk5-mediated phosphorylation can play either a positive or negative role in synaptic formation and function. For example, the Cdk5-dependent phosphorylation of tropomyosin receptor kinase in response to brain-derived neurotrophic factor is important for promoting the formation of the dendritic spines in the hippocampus [46], while Cdk5-dependent phosphorylation of the Rho guanine nucleotide exchange factor ephexin1 or Wiskott–Aldrich syndrome protein-family protein WAVE1 reduces the number of dendritic spines through the modulation of actin [47]. Rho GTPases are key regulators of the actin cytoskeleton and are important in the organization and remodeling of dendritic spines [44]. WAVE1 localizes to dendritic spines and contributes to the formation of spines, as well as to the remodeling of the actin cytoskeleton in spines during development and other processes affecting synaptic plasticity [48]. WAVE1 is a physiological substrate for Cdk5, and its phosphorylation at three sites is dependent on the activity of Cdk5 [48]. Loss of WAVE1 in vivo and in cultured neurons results in a decrease in mature dendritic spines [47, 48]. This decrease in mature spines suggests a possible role for Cdk5-dependent phosphorylation of WAVE1 in the formation of the actin cytoskeleton in neurons, and consequently, in the regulation of dendritic spine morphology.

To conclude, our present study demonstrates novel insights into the multifaceted actions of CRF, its receptors presence and localization in CA1, and intricate molecular mechanisms that regulate spine dynamics in response to acute stress and contributes to the better understanding of the intricate interplay between neuropeptide-driven signaling and synaptic plasticity.

### Supplementary information

Vandael et al_Sup_Information

Vandael et al_Sup_Figure 1

## Data Availability

The data that support the findings of this study are available from the corresponding author upon reasonable request.

## Material availability

The materials that support the findings of this study are available from the corresponding author upon reasonable request.
