# Supplementary material for: Cdk5-dependent rapid formation and stabilization of dendritic spines by corticotropin-releasing factor
Source: Transl Psychiatry. 2024 Jan 17;14:29. doi: 10.1038/s41398-024-02749-7 (PMC10794228; doi:10.1038/s41398-024-02749-7)
Supplement: Supplementary file 1 — Vandael et al_Sup_Information [file 41398_2024_2749_MOESM1_ESM.docx]

## **SUPPLEMENTARY INFORMATION**

**Materials and Methods**

For the Tokuyasu immunolabeling method, C57BL/6Jax mice were perfused with 2% PFA + 0.01% GA in 0.1M PB and stored in the same fixative overnight. The following day, specimens were subjected to a series of sample preparation steps. This involved three subsequent cycles of immersion in 0.1M PB for 10 minutes. Subsequently, vibratome sections were prepared with a thickness of 150µm. The brain sections were flat embedded in 12% gelatin, and the specific regions of interest in the CA1-stratum radiatum (SR) were excised. Each cropped sample was cryoprotected by infiltration with a solution of 2.3 M sucrose in 0.1 M PB (pH 7.4) at 4 °C overnight. The sucrose-infiltrated CA1-SR samples were then mounted on aluminum pins and immediately submerged into liquid nitrogen and stored until further processing. The cryoprotected pins with frozen brain tissue were used for cryo-ultramicrotome cutting with a UC7 Ultratome and cryo FC7 unit (Leica). Tissue blocks were trimmed at −90 °C with a cryotrim diamond knife (Diatome) and ultrathin cryosections (70-100 nm) were cut at −110 °C with an immune diamond knife (Diatome). Sections were picked up with a drop containing an equal mixture of 2% methylcellulose (Sigma-Aldrich) and 2.3 M sucrose (25), warmed up to RT, and transferred onto a formvar film-coated 100-mesh copper grid (EMS). For immune-gold labeling of CRF and CRF-R1, we used the following specific antibodies and dilution ratios in our studies: primary 1:1000 rabbit anti-CRF (Salk), 1:200 rabbit anti-CRF-R1 (ACR-050, Alomone labs), 1:200 goat anti-VGluT1 (VGluT1-Go-Ad310, Frontier Institute co.,ltd), and secondary1:30 donkey anti-rabbit 6 nm gold and 1:20 donkey anti-goat 10nm gold (Aurion). Initially, we performed single labeling with CRF (6mn) or employed double labeling (for recognizing pre- and postsynaptic sites) with CRF-R1 (6nm) and VGluT1 (10nm). Subsequently, the grids were incubated in 50 mM glycine in phosphate-buffered saline (PBS) for 15 minutes, followed by incubation in a protein-blocking solution of 5% bovine serum albumin (BSA, EMS), 5% donkey serum (Jackson Laboratory), and 0.1% cold water fish gelatin (EMS) in PBS for 30 minutes. After the blocking step, the grids underwent three cycles of washing with 0.1% BSAc (Aurion) in PBS for 5 minutes each and incubated with the first primary antibodies in the same solution at 4 °C overnight. The following day, the grids were washed by passing a series of droplets of 0.1% BSAc in PBS. Afterward, the grids were incubated with secondary antibodies for 2 hours at RT in the same solution. Subsequently, the grids were washed again with 0.1% BSAc in PBS, followed by incubation of the second primary antibody in 0.1% BSAc in PBS at 4°C overnight. The next day, after washing with 0.1% BSAc in PBS, the grids were incubated with the second secondary antibodies for 2 hours at RT in the same solution. Lastly, the grids were washed with 0.1% BSAc in PBS followed by rinsing in pure PBS. Then, the grids were fixed with 1% GA in PB for 10 minutes at RT. After the final wash in PBS and distilled water, the grids were transferred on drops of 1.8% methylcellulose and 0.4% uranyl acetate (EMS) for 8 minutes. Finally, the grids were removed, and most of the excess viscous solution was drained. The sections were air-dried, leaving a thin film that remained over the grid, enabling direct EM observation with a JEM1400 TEM (Jeol) equipped with an SIS Quemesa (Olympus) camera operating at 80 kV.

**SUPPLEMENTARY FIGURE 1 LEGEND**

**The Presence CRF and CRF-R1 in the SC-CA1 hippocampal synapse.** (A, left) TEM images of CA1-SR synapses. Immunogold (6nm) labeling for CRF is present in the interneuronal (IN, left) and excitatory (right) synapses at both parts of the synaptic boutons (PRE: presynaptic part of the synapse, POST: postsynaptic part of synapse). (A, right) Double labeling for VGLUT1 (10nm, white arrows) and CRF (6nm, black arrows) confirms that labeling is predominantly located in the presynaptic compartment. (B, left) TEM images of CRF-R1 (6nm) immunogold labeling. CRF-R1 appears at both synaptic sites in excitatory synapses, as confirmed (B, right) with double labeling for CRF-R1 (6nm, black arrows) and VGLUT1 (10nm, white arrows). Scale bar=250 nm.
