# Supplementary figures and images for: Cdk5-dependent rapid formation and stabilization of dendritic spines by corticotropin-releasing factor
Source: Transl Psychiatry. 2024 Jan 17;14:29. doi: 10.1038/s41398-024-02749-7 (PMC10794228; doi:10.1038/s41398-024-02749-7)

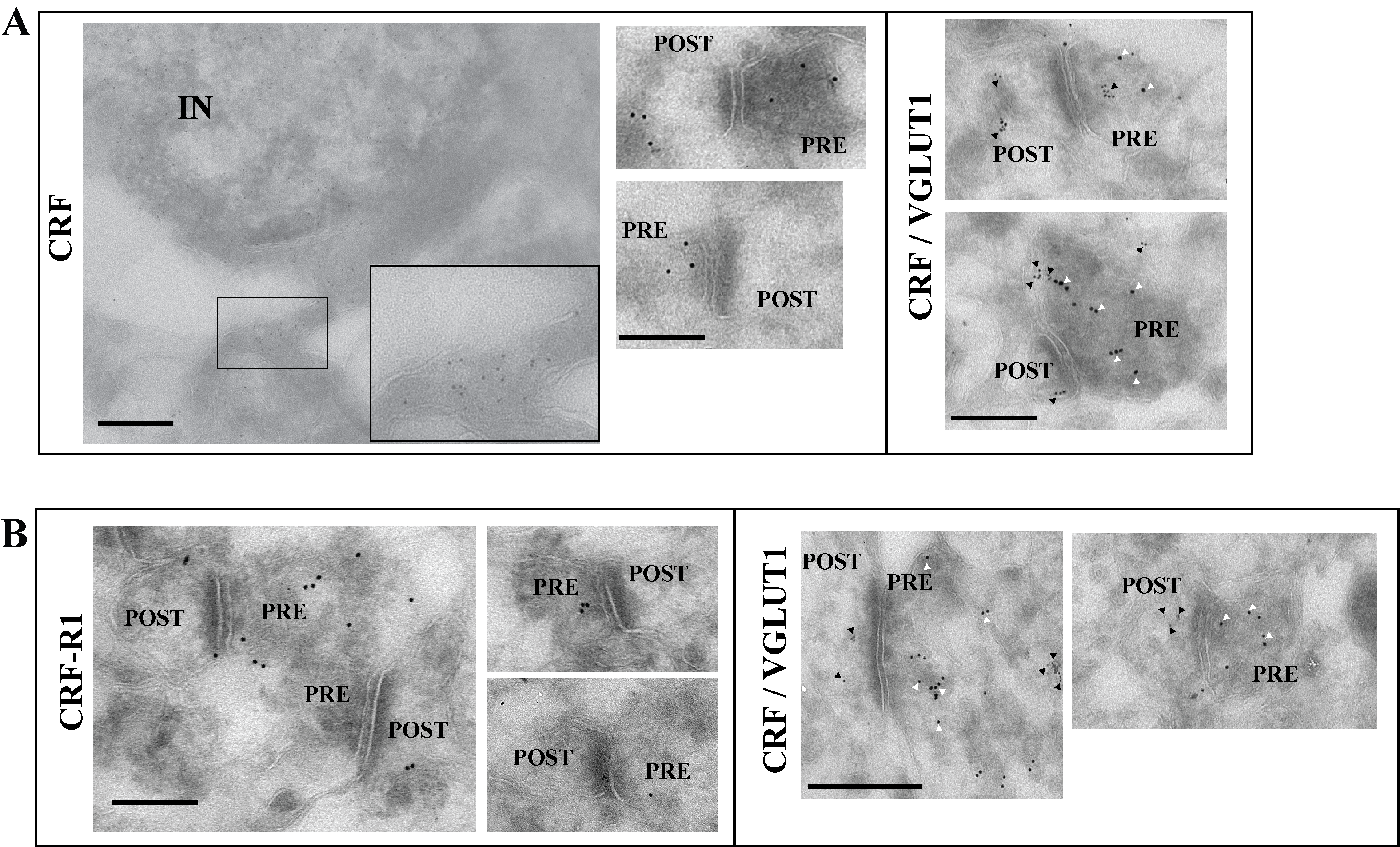

Supplement: Supplementary file 2 — Vandael et al_Sup_Figure 1 [file 41398_2024_2749_MOESM2_ESM.tif]
